# PDeT: A Progressive Deformable Transformer for Photovoltaic Panel Defect Segmentation

**DOI:** 10.3390/s24216908

**Published:** 2024-10-28

**Authors:** Peng Zhou, Hong Fang, Gaochang Wu

**Affiliations:** 1 State Key Laboratory of Synthetical Automation for Process Industries, Northeastern University, Shenyang 110819, China; 2 School of Artificial Intelligence and Data Science, Hebei University of Technology, Tianjin 300130, China; 3 School of Information and Electronic Engineering, Zhejiang Gongshang University, Hangzhou 310018, China

**Keywords:** photovoltaic panel defects, defect segmentation, deformable attention, feature aggregation

## Abstract

Defects in photovoltaic (PV) panels can significantly reduce the power generation efficiency of the system and may cause localized overheating due to uneven current distribution. Therefore, adopting precise pixel-level defect detection, i.e., defect segmentation, technology is essential to ensuring stable operation. However, for effective defect segmentation, the feature extractor must adaptively determine the appropriate scale or receptive field for accurate defect localization, while the decoder must seamlessly fuse coarse-level semantics with fine-grained features to enhance high-level representations. In this paper, we propose a Progressive Deformable Transformer (PDeT) for defect segmentation in PV cells. This approach effectively learns spatial sampling offsets and refines features progressively through coarse-level semantic attention. Specifically, the network adaptively captures spatial offset positions and computes self-attention, expanding the model’s receptive field and enabling feature extraction across objects of various shapes. Furthermore, we introduce a semantic aggregation module to refine semantic information, converting the fused feature map into a scale space and balancing contextual information. Extensive experiments demonstrate the effectiveness of our method, achieving an mIoU of 88.41% on our solar cell dataset, outperforming other methods. Additionally, to validate the PDeT’s applicability across different domains, we trained and tested it on the MVTec-AD dataset. The experimental results demonstrate that the PDeT exhibits excellent recognition performance in various other scenarios as well.

## 1. Introduction

Defects in photovoltaic (PV) cell substrates can reduce photoelectric conversion efficiency, leading to a decrease in system power generation [1]. These defects may also cause uneven current distribution in localized areas, resulting in hotspot effects [2]. This abnormal temperature increase in defect regions not only further decreases power generation efficiency but can also damage PV panels and even pose safety hazards, such as fire risks. Therefore, incorporating defect detection technology in PV quality control processes is crucial for identifying and preventing such defects in advance, ensuring the efficient and safe operation of PV systems. Defect segmentation (pixel-level defect detection) [3] provides detailed information about defects, including their precise location, shape, and size. With accurate segmentation data, PV manufacturers can detect potential issues in the panels early, preventing further problems and losses during the system’s operational phase [4]. This not only boosts the economic benefits for enterprises but also contributes to the sustainable development of clean energy.

Recent research has utilized deep learning-based segmentation models [5,6,7,8,9,10,11,12,13,14,15] to segment defects in solar panels. Compared to manual inspection, deep learning techniques offer significant advantages. First, deep learning can automatically process and analyze large amounts of data, greatly improving inspection efficiency, especially in large-scale production settings. Second, deep learning models exhibit high consistency and precision in identifying complex features, avoiding fatigue and subjective errors that are common in manual inspections. Additionally, deep learning can continuously optimize and improve through data training, adapting to new types of defects and changing environments, which manual inspection cannot match in terms of adaptability and continuous improvement.

However, current defect segmentation algorithms still struggle with detecting elongated cracks and tend to miss or falsely detect small flaws around grid lines. During the feature extraction stage (encoding phase), Convolutional Neural Networks (CNNs) are limited by their fixed position sampling of input features. This constraint makes it difficult for models to handle fine-grained recognition tasks like detecting elongated cracks, leading to confusion or the loss of important features [3]. Although Vision Transformers (ViTs) [16] have been proven to effectively capture long-range feature relationships, the dense attention mechanism in ViTs results in high memory and computational costs. In the feature fusion and upsampling stage (decoding phase), the transmission of high-level semantic information to shallow layers gets disrupted and diluted by a large number of local patterns in the shallow layers [17].

Therefore, learning-based methods for PV defect segmentation need to address the following challenges. First, in the feature extraction phase, the encoder needs to adaptively select feature sampling locations. Second, in the feature fusion and upsampling phase, the decoder must refine features based on coarse-level semantics and prevent the dilution of fine-grained information.

In this paper, we propose a novel Progressive Deformable Transformer, dubbed PDeT, to effectively learn the offset of spatial sampling positions and progressively refine features based on coarse-level semantics for enhancing defect segmentation in PV panels. Specifically, we employ a deformable self-attention module in the encoder, which generates reference points as a unified network and takes query features as input for learning offsets, generating corresponding offsets for all reference points. In this way, the candidate keys/values can focus on defect regions and other crucial areas, enabling precise localization of defect features in complex backgrounds. This module is more flexible and efficient, allowing the dynamic adjustment of attention regions, thereby capturing the edges and details of defects more effectively and improving the accuracy of segmentation. In the decoder, we introduce a semantic aggregation module that combines feature differences across four different scales, reassembling coarse-level features with fine-grained ones. This approach effectively distinguishes between background and defect features in PV panels, providing clear and detailed spatial boundaries without losing semantic information. Our PDeT is capable of flexibly adjusting spatial sampling positions and progressively refining feature extraction using coarse-level semantic information, thereby improving the accuracy and reliability of PV defect detection.

The main contributions of this paper are summarized as follows:

- A progressive deformable Transformer is proposed to achieve high-quality segmentation of PV panel defects, significantly enhancing the ability to detect complex defect features.
- A deformable self-attention module is introduced to adaptively learn spatial feature sampling locations. This module adjusts sampling positions based on the shape and structure of input features, flexibly capturing irregular or deformed features.
- A semantic aggregation module is designed to ensure the retention and integration of both coarse and fine-grained information, effectively balancing these features while incorporating contextual information for better segmentation accuracy.

We conducted extensive experiments to validate the effectiveness of the introduced modules. On the solar cell defect dataset, our model achieved 88.41% mIoU. Additionally, we also conducted training and evaluation on the MVTec-AD [18] dataset. The experimental results indicate that the PDeT demonstrates exceptional recognition capabilities across different scenarios.

## 2. Related Work

This paper focuses on photovoltaic defect segmentation using a Vision Transformer backbone. To provide context, we present a brief overview of relevant research in this area.

### 2.1. Photovoltaic Defect Segmentation

The photovoltaic industry has extensively adopted deep learning-based methods for defect detection, offering significant improvements in both detection efficiency and accuracy compared to traditional approaches. By analyzing features from large-scale datasets, deep learning enables the precise identification of defects in photovoltaic panels. Tang et al. [15], for instance, used limited samples to generate high-resolution electroluminescence images and employed CNNs to automatically classify defects. Jiang et al. [13] proposed an m-shaped architecture to extract and fuse shallow and deep features in segmentation networks, incorporating an attention module to suppress the photovoltaic background and improve segmentation accuracy. Xie et al. [12] embedded a domain discriminator into the CNN to distinguish between data domains, allowing the feature extractor to learn domain-invariant features. Pratt et al. [19] evaluated four deep learning models (U-Net, PSPNet, and DeepLabv3+) for detecting cracks, inactive areas, and grid line defects in photovoltaic panels. Jha et al. [11] introduced a semi-supervised semantic segmentation method for defect detection with limited labeled data, significantly reducing manual annotation costs. Kaligambe et al. [10] developed a lightweight CNN model and compared its performance against a fine-tuned VGG16 model. Fioresi et al. [8] used ResNet50 and DeepLabv3 as backbone networks and segmentation decoders to identify defects such as cracks, contact interruptions, cell interconnection failures, and contact corrosion in both polycrystalline and monocrystalline silicon cells. Chen et al. [7] fine-tuned a U-Net model with a pre-trained VGG16 encoder, achieving excellent segmentation results for cracks and busbars. Similarly, Zhang et al. [6] refined CNN-extracted features using a global pairwise similarity module and a connection saliency module, enhancing defect detection in photovoltaic images.

### 2.2. Vision Transformer

The Vision Transformer (ViT) [16] has revolutionized computer vision tasks by leveraging the self-attention mechanism originally designed for natural language processing. By treating images as sequences of patches, ViT captures global context more effectively than traditional convolutional neural networks (CNNs). However, the high computational complexity and large data requirements have led to the development of more efficient Transformer variants. One such model is the Data-Efficient Image Transformer (DeiT), introduced by Touvron et al. [20], which improves the training process of ViT through data augmentation and advanced techniques, making it more suitable for smaller datasets.

In addition to image classification, ViT has been successfully applied to tasks like object detection and segmentation [21,22]. To enhance Transformer-based architectures further, Liu et al. [23] developed the Swin Transformer, which utilizes a hierarchical structure and local window self-attention, significantly improving computational efficiency and achieving state-of-the-art performance across multiple benchmarks.

Beyond these advancements, the neighborhood attention Transformer [24] offers an alternative approach by refining local attention mechanisms. Unlike the standard self-attention mechanism that emphasizes global information, NAT focuses on attention within local neighborhoods, making it particularly effective for tasks requiring fine-grained feature extraction. In summary, while ViTs laid the foundation for Transformer-based models in computer vision, optimizations in models like the DeiT, Swin Transformer, and neighborhood attention Transformer have addressed challenges such as computational complexity and fine feature extraction, broadening their applicability to a wider range of visual tasks.

## 3. Methodology

In this section, we delve into the specific architecture of the proposed Progressive Deformable Transformer (PDeT) designed for photovoltaic panel defect segmentation. The ViT [16] was the first to demonstrate the viability of Transformer mechanisms in computer vision. Unlike CNNs, Transformer-based models, with their Multi-Head Self-Attention (MHSA) mechanism, excel at capturing long-range dependencies, making them particularly effective for target localization. Building on this foundation, we introduce a deformable sampling operation before the MHSA in each Transformer encoder. This operation enables the network to focus more effectively on relevant regions. In the decoder, we propose a semantic aggregation module, which enhances the fusion of multi-stage features, improving overall segmentation accuracy. The following subsections will provide a detailed explanation of the network framework, the Deformable Transformer Block, and the Semantic Aggregation Block.

### 3.1. PDeT Overall Architecture

**Figure 1:**
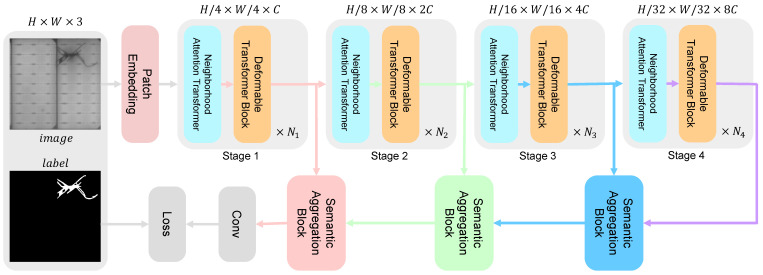
Overall architecture of the proposed PDeT for photovoltaic defect segmentation.

Our PDeT follows the widely used encoder–decoder structure. The encoder extracts feature information from the input image, while the decoder progressively restores the spatial dimensions to generate pixel-level segmentation results. As illustrated in Figure 1, the input image of shape H×W×3 is first processed through two convolutional and normalization embedding layers, producing patch embeddings of size H/4×W/4×C (where C=128). Each convolution employs a kernel size of 3×3, with a stride of 2 and padding of 1, expanding output channels from 3 to 64 and then to 128. To construct a multi-level feature pyramid, our backbone is organized into four stages, each with a progressively increasing stride. Each stage comprises *N* stacked neighborhood attention Transformer blocks (NATs) [24] and Deformable Transformer Blocks (DTBs). In our implementation, $\left\{N_{1},N_{2},N_{3},N_{4}\right\}$ are set to $\left\{1,2,9,1\right\}$, respectively. The feature maps extracted at each stage are downsampled by a factor of 4, 8, 16, and 32 times the input size, denoted as $f=\left\{f_{i}|1\leq i\leq 4\right\}$.

Next, we aim to seamlessly fuse and upsample the multi-level features in the decoder stage. We employ the Semantic Aggregation Block (SAB) to fuse adjacent features $\left\{f_{i},f_{i+1}\right\}$, with the output serving as the input to the subsequent SAB. This process reduces inconsistencies between feature levels, allowing coarse-level semantic information to effectively refine finer features, thereby achieving a balance between global semantics and local details. The output from the encoder is processed through two rounds of bilinear interpolation and convolution operations to generate the predicted output.

### 3.2. Deformable Transformer Block

Before introducing deformable attention into the Transformer, we will first review the vanilla MHSA mechanism in the Transformer. Given an input feature map $x\in R^{H\times W\times C}$, the MHSA mechanism with *M* attention heads is applied, where each attention head independently learns different relationships. The outputs from these attention heads are then concatenated. The vanilla MHSA mechanism can be formulated as follows:

(1) $$\begin{array}{cc} q & =xW_{q},k=xW_{k},v=xW_{v},\\ y^{m} & =\mathrm{softmax}\left(\frac{q^{\left(m\right)}k^{\left(m\right)T}}{\sqrt{d}}\right)v^{\left(m\right)},m\in \left\{1,\ldots ,M\right\},\\ y & =\mathrm{concat}y^{\left(1\right)},\ldots ,y^{\left(M\right)}W_{o}, \end{array}$$

where $d=\frac{C}{M}$ represents the dimension of each attention head, and $q^{\left(m\right)},k^{\left(m\right)},v^{\left(m\right)}$ denote the query, key, and value embeddings, respectively. The output of the *m*-th attention head is represented as $y^{\left(m\right)}$. The learnable parameters $W_{q},W_{k},W_{v},W_{o}\in R^{C\times C}$ are the projection matrices for the feature embeddings. The formulation for the *i*-th Transformer block is given as follows:

(2) $$\begin{array}{cc} y_{i}^{\prime } & =\mathrm{MHSA}\left(\mathrm{LN}(y_{i-1})\right)+y_{i-1},\\ y_{i} & =\mathrm{MLP}\left(\mathrm{LN}(y_{i}^{\prime })\right)+y_{i}^{\prime }, \end{array}$$

where LN(·) represents Layer Normalization [25], MHSA(·) denotes the MHSA mechanism in Equation (1), and MLP(·) is the MLP with two linear layers.

Despite the vanilla MHSA mechanism’s effectiveness in capturing global information, it has certain limitations when processing local features and irregular shapes. To address these issues, we incorporate deformable operations into our PDeT. Deformable operations allow flexible attention adjustments, enabling the model to better focus on critical information. This is especially effective for handling complex or irregular features in PV defect segmentation. Next, we will provide a detailed explanation of how deformable operations are integrated into MHSA to enhance the performance of Transformer models.

**Figure 2:**
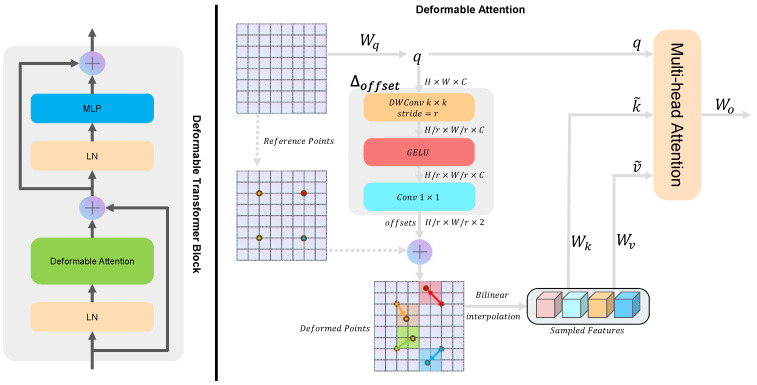
Detailed structure of the Deformable Transformer Block (DTB).

As shown in Figure 2, our goal is to enable the key–value pairs to adaptively sample feature information based on offsets provided by the offset network. First, given the input feature $x\in R^{H\times W\times C}$, a uniform grid of reference points $r\in R^{H_{r}\times W_{r}\times 2}$ is generated. This grid is downsampled by a factor of relative to the size of the input feature, where $H_{r}\in \frac{H}{r}$ and $W_{r}\in \frac{W}{r}$. The values of the reference points correspond to 2D coordinates $\left(0,0\right),\ldots \left(H_{r}-1,W_{r}-1\right)$. The ranges of the two dimensions, $\left[0,\left(H_{r}-1\right)\right]$ and $\left[0,\left(W_{r}-1\right)\right]$, are then normalized to [−1,+1], where $\left(-1,-1\right)$ represents the reference point at the top-left corner and $\left(+1,+1\right)$ represents the reference point at the bottom-right corner.

Next, we need to obtain the offsets for each reference point. We start by using $W_{q}$ to generate the query tokens *q*, which are then input into the $\Delta_{offset}$ module to learn the offsets for the reference points. This module consists of a depth-wise convolution, a GELU activation function, and 1×1 convolution, resulting in an output with the same shape as the reference points. The calculated offsets are then added to the reference points to derive the positions of the deformable points. Sampling is performed at these deformable points, and the keys and values are computed using the projection matrices. At this stage, we obtain the deformable keys $\tilde{k}$ and values $\tilde{v}$, which are combined with *q* to compute the MHSA. Consequently, Equation (1) is updated to:

(3) $$\begin{array}{cc} q & =xW_{q},\tilde{k}=\tilde{x}W_{k},\tilde{v}=\tilde{x}W_{v}\\ \Delta_{p} & =\Delta_{offset}\left(q\right),\\ \tilde{x} & =\varphi (x;p+\Delta_{p}), \end{array}$$

where $\Delta_{p}$ represents the offset of the reference point, **p** represents the position of the reference point, and φ(a;b) denotes the sampling result at location b in the feature map *a*.

This deformable operation enables the Transformer structure to adaptively sample based on the query features, allowing the model to flexibly adjust the shape and position of the self-attention, particularly when handling features that span large scales. Moreover, by integrating the deformable operation, the only component that introduces a small number of learnable parameters is the $\Delta_{offset}$ module. This is one reason why we employ lightweight depth-wise convolution, ensuring that the model remains efficient while maintaining performance.

### 3.3. Semantic Aggregation Block

**Figure 3:**
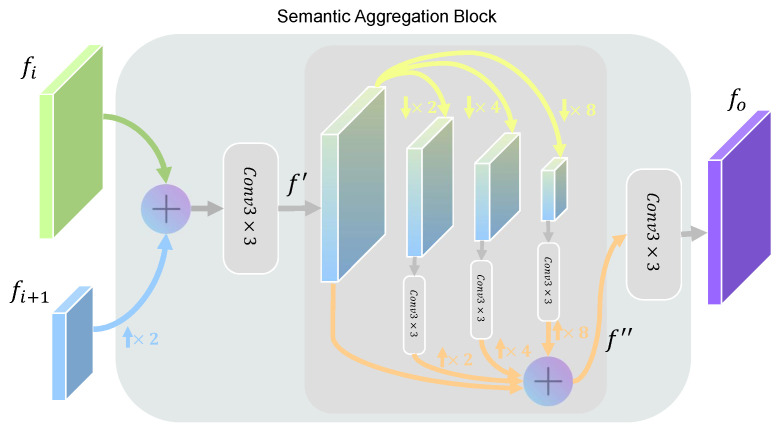
Detailed structure of the Semantic Aggregation Block (SAB).

The encoder of our PDeT incorporates multiple stages to generate feature maps at different hierarchical levels. To progressively fuse the feature maps extracted from each stage, we integrate the Semantic Aggregation Block (SAB), which extracts and merges features across these levels. This block effectively preserves coarse semantic information while retaining fine-grained details, ultimately leading to high-quality segmentation results. Figure 3 illustrates the detailed structure of the SAB.

The SAB processes feature maps from two different stages, $f_{i}$ and $f_{i+1}$, where i∈{1,2,3}. We first align the channel dimensions of the feature maps into C=256 via a 1×1 convolution. Then, a bilinear interpolation is applied to upsample the higher-level semantic feature $f_{i+1}$, resizing it to match the dimensions of the lower-level feature $f_{i}$. These operations lay the groundwork for subsequent feature fusion. The interpolated feature is then added to the lower-level feature, followed by a 3×3 convolution layer for initial feature extraction. This step not only enhances the fused feature representation but also captures richer detail information. The process is formulated as:

(4) $$f^{\prime }={\mathrm{Conv}}_{3\times 3}\left(f_{i}+\phi_{bi}\left(f_{i+1};2\right)\right),$$

where $\phi_{bi}\left(\cdot ;2\right)$ indicates the 2×2 bilinear upsampling operation, ${\mathrm{Conv}}_{3\times 3}$ represents the 3×3 convolution layer, and $f^{\prime }$ is the intermediate feature map within the SAB.

Next, the SAB applies an average pooling operation at multiple scales, transforming the feature map into various spatial resolutions. This multi-scale processing allows the model to capture features comprehensively, from local details to global context. It enhances the model’s ability to perceive defect regions at different scales and improves its adaptability to diverse scenes and variations. After convolutional processing, the features from each sub-branch are upsampled to the original size and combined with the input feature map through residual connections. These connections ensure that original feature information is not lost during multi-scale transformations, preserving both fine-grained details and global semantic information, thereby enhancing the stability and expressiveness of the fusion. The process can be formulated as:

(5) $$f^{\prime \prime }=f^{\prime }+\sum_{j\in \{2,4,8\}}\phi_{bi}\left({\mathrm{Conv}}_{3\times 3}\left(\mathrm{Pool}(f^{\prime };j)\right);j\right),$$

where Pool(·;j) indicates the average pooling operation at scale j∈{2,4,8}.

Finally, a 3×3 convolution layer is applied to the fused features for further refinement and smoothing, generating the output of the SAB as $f_{o}={\mathrm{Conv}}_{3\times 3}\left(f^{\prime \prime }\right)$. This step enhances the features after multi-scale processing, improving the model’s contextual awareness and leading to more robust feature representations.

Overall, the proposed SAB achieves a balance between coarse and fine-grained information through multi-scale processing, enabling the model to generalize effectively when dealing with targets of varying scales, shapes, and distributions, thereby improving its adaptability and expressive power in complex photovoltaic environments.

## 4. Experiments

We conducted ablation and comparison experiments on the photovoltaic dataset to validate the performance of the PDeT network. Additionally, to assess the applicability of the PDeT network in other industrial scenarios, we selected four industrial scenes from the public MVTec-AD [18] dataset for further evaluation.

### 4.1. Implementation

All experiments were conducted using an NVIDIA GeForce RTX 3060 (12GB) and an Intel Core i5-12490F processor, both sourced from Santa Clara, CA, USA. The model was built and trained using the PyTorch 1.12.0 framework. During training, the AdamW [26] optimizer was employed with an initial learning rate of 0.0001 and a weight decay coefficient of 0.0001. The exponential decay rates for computing the first- and second-moment estimates of gradients were set to 0.9 and 0.999, respectively. A polynomial learning rate schedule (with a power of 0.9) was adopted, allowing dynamic adjustment of the learning rate throughout training. The maximum number of iterations was set to 160,000, with model evaluations performed every 8000 iterations using the mIoU as the evaluation metric. Cross-entropy loss was used to calculate the loss, ensuring efficient model convergence and accurate classification.

The photovoltaic dataset used in our experiments contains a total of 1024 images, which were split into training and validation sets at a 2:1 ratio. Each image has a resolution of 640×640, and the segmentation results are classified into two categories: defect and background. During training, we applied random cropping as a data augmentation technique to both the input images and their corresponding labels.

### 4.2. Ablation Study

We conducted ablation experiments using the photovoltaic panel dataset. To validate the effectiveness of the deformable convolution, we sequentially replaced the NAT Block in the feature extraction stage with the DTB. In the overall network structure described in Section 3, we stack $N_{i}$ Transformer blocks in each stage. Each Transformer block originally consisted of two NATs in series as the baseline, and then, we replaced the second NAT in each Transformer with the DTB. The results, as shown in Table 1, indicate that while adding the DTB only in the fourth stage slightly decreases model performance, incorporating the DTB in the third stage yields an improvement of about one percentage point. When all stages utilize the DTB, the model performance increases by approximately three percentage points compared to the baseline.

As shown in Figure 1, we replaced the SAB with standard convolution and bilinear interpolation to establish a comparison baseline, effectively omitting the transition from $f^{\prime }$ to $f^{\prime \prime }$. We then sequentially integrated multi-scale methods into the different stages of the decoder. As shown in Table 2, using SAB in stages 1 and 2 resulted in a performance improvement of 0.62%, while adding SAB from stages 2 to 3 further increased performance by 1.12%.

To evaluate the impact of different Decoder heads on model performance while keeping the encoder architecture unchanged, we conducted comparative experiments. This experiment utilized the encoder proposed in this paper as the baseline architecture and introduced various Decoder heads to observe their performance in specific tasks. The experimental results, as shown in Table 3, indicate that the IoU for the background class remained consistent across all Decoder heads. However, significant differences were observed in the IoU for the defect class among the various Decoder heads.

Specifically, the IoU for the defect class with the FPN [27] head is 74.52%, resulting in an overall mIoU of 87.25%. The UPer [28] head shows a slight improvement, with a defect class IoU of 75.36% and an overall mIoU of 87.67%. The most significant enhancement is observed with the proposed PDeT model I, achieving a defect class IoU of 76.85% and an overall mIoU of 88.41%. This indicates that the PDeT demonstrates advantages in fine-grained feature aggregation when processing defect features, significantly improving the model’s performance in defect detection tasks.

**Figure 4:**
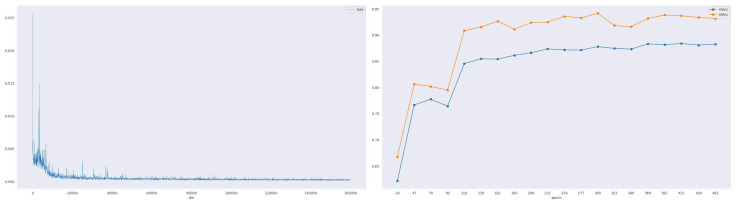
The training process of the PDeT is assessed using three metrics: loss, mIoU, and mAcc. These metrics offer valuable insights into the model’s performance and effectiveness during training. The left panel displays the loss at each iteration, while the right panel presents the validation results throughout the training process.

Figure 4 illustrates the training process of the PDeT model. During the initial phase of training, the loss fluctuates significantly, indicating that the model is still learning and adjusting its parameters. However, as training progresses, the model gradually learns to capture the characteristics of the data, leading to a steady improvement in both the overall mIoU and mAcc metrics. Notably, the best mIoU is achieved at the 300th epoch, reaching 88.41%, which reflects the model’s superior performance at this stage. Meanwhile, the loss value gradually converges and stabilizes at a lower level, indicating that the model effectively reduced its error rate during training.

### 4.3. Comparison with Other Segmentation Networks

In this section, we compare our method with existing semantic segmentation networks, including EMANet [29], STDC [30], DDRNet [31], K-Net [32], DNLNet [33], CCNet [34], ANN [35], DMNet [36], PIDNet [37], ISANet [38], GCNet [39], and SIIF [3].

Table 4 provides a detailed comparison of various models in the task of defect detection on photovoltaic panels, including metrics such as model parameters (Params), floating-point operations per second (FLOPs), frames per second (FPS), precision, recall, F1 score, and mIoU. Precision represents the proportion of true positive samples among those predicted as positive by the model. High precision indicates that most of the detected defects are genuine. Recall, on the other hand, measures the proportion of correctly identified positive samples out of all actual positive cases. High recall signifies that the majority of actual defects have been successfully detected. Additionally, mIoU reflects the model’s ability to distinguish between defect areas and the background. A higher mIoU ensures greater detection accuracy and fewer missed defects.

Analysis reveals that the PDeT model outperforms others by at least four percentage points in mIoU, demonstrating its exceptional performance in photovoltaic defect segmentation. This advantage is primarily due to PDeT’s feature extraction network, which employs a Transformer architecture. However, this comes at the cost of a relatively large number of parameters, leading to increased computational overhead. Thus, there is still room for optimization in the inference efficiency of the PDeT. In contrast, models like DDRNet-S and STDC, while having fewer parameters and higher computational efficiency, generally perform worse in the actual segmentation results, showing a significant gap compared to the PDeT. Despite their successes in lightweight design, these models exhibit relatively limited feature extraction capabilities when dealing with complex defect backgrounds, making it challenging to accurately capture the details of defect regions.

**Figure 5:**
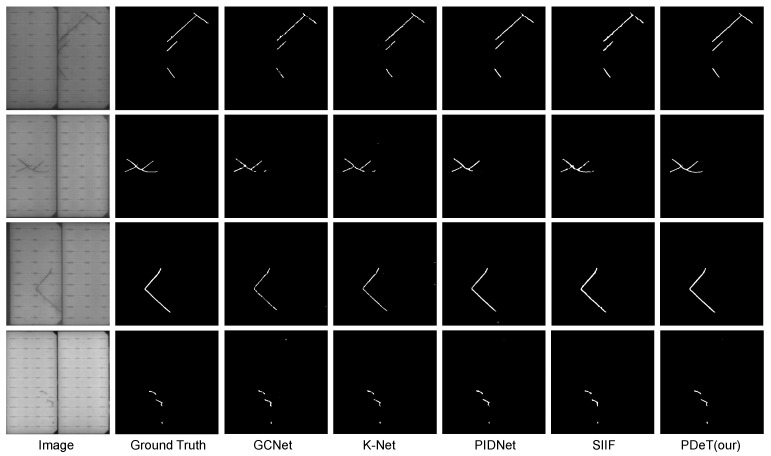
Quantitative comparison with other segmentation networks.

Figure 5 presents a detailed comparison of different networks in the segmentation of defects on photovoltaic panels. By examining the results, we can clearly observe the differences among models when addressing specific types of defects, particularly in the segmentation of elongated cracks, where the discrepancies are especially pronounced. From the segmentation results in the first and third rows, it is evident that many models struggle with elongated cracks, often producing coarse results. These defects typically exhibit fine, elongated features, and conventional segmentation networks tend to overlook edge information when dealing with such detail-rich defects, leading to imprecise segmentation outcomes. For instance, while K-Net, a model with a larger number of parameters, has strong global perception capabilities during feature extraction, allowing it to capture the general outline of defects, the edges of the cracks in its output still appear blurred, failing to accurately segment the detailed parts of elongated defects.

In contrast, the PDeT demonstrates superior capability in handling such defects. The model employs deformable self-attention, which allows for dynamic adjustment of sampling point positions, enabling a more flexible focus on the edges of cracks and, thus, accurately capturing these details. Moreover, during the decoding process, the SAB module plays a crucial role in refining segmentation. This module progressively aggregates coarse and fine feature information by fusing multi-scale features, allowing for precise segmentation of defect edges without losing semantic information. Due to the need for further optimization of the model’s parameter count and computational load, we will consider employing pruning and quantization methods suitable for Transformer architectures in the future. These approaches aim to reduce the model’s computational cost and memory usage. Figure 5 clearly illustrates that the PDeT’s segmentation results outperform other models in detailing and edge treatment of cracks. This refined segmentation ability enables the PDeT to maintain global perception while flexibly adjusting its focus areas and enhancing the feature aggregation process, ultimately improving defect detection accuracy.

### 4.4. Applicability in Other Industrial Fields

To validate the application potential of the PDeT photovoltaic defect detection model in other industrial scenarios, we selected four typical scenes from the MVTec-AD [18] anomaly detection dataset for experimentation. These scenes include Wood, Metal Nut, Tile, and Hazelnut, representing common complex backgrounds and diverse defect types in industrial manufacturing, thus providing high representativeness. The selection of these scenes aims to ensure that the PDeT model demonstrates good adaptability across different materials and defect patterns, further validating its broad potential for practical industrial applications.

The MVTec-AD dataset is primarily used for anomaly detection and contains both normal and abnormal data. In our experimental design, we performed stratified sampling for each scene, dividing the normal and abnormal data into training and testing sets at a 2:1 ratio. This approach simulates real industrial defect detection tasks and aligns more closely with actual application requirements. The evaluation metric for the model is mIoU, which is widely used in semantic segmentation tasks and provides a comprehensive assessment of the model’s recognition performance across various categories.

In this experiment, we compared the performance of K-Net, UPerNet, GCNet, and SIIF models across four scenes in the MVTec-AD dataset. The experimental results are presented in Table 5, with each column representing the mIoU for the respective scenes.

The performance of K-Net [32] across the four scenes is relatively low, with an mIoU of 76.06% for Hazelnut and 74.17% for Wood, indicating its limited recognition capability in complex backgrounds. In contrast, UPerNet [28] performed exceptionally well in all scenes, achieving mIoU of 93.57% and 96.6% for Hazelnut and Metal Nut, respectively, demonstrating its superior feature extraction ability. The performances of GCNet [39] and SIIF [3] are comparable to UPerNet, maintaining high mIoU values across the scenes. Notably, in the Metal Nut scene, GCNet [39] achieved an mIoU of 95.32%, indicating a certain advantage in detecting defects in metal nuts. It is noteworthy that the proposed PDeT model performed well across all four scenes: Hazelnut, Metal Nut, Tile, and Wood. The mIoU for the Hazelnut scene was 94.34%, while for the Metal Nut scene, it was 95.32%. This indicates that the PDeT model demonstrates strong adaptability and stability across different materials and defect patterns.

**Figure 6:**
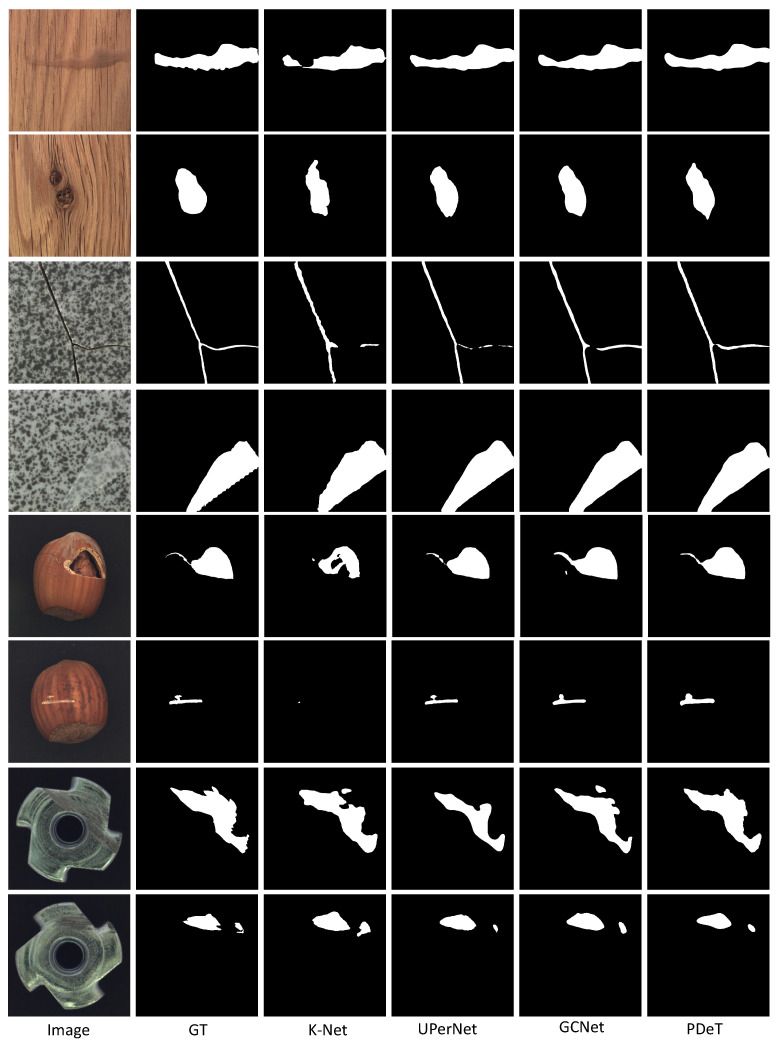
Quantitative display of segmentation results from multiple models across four distinct scenes, with each scene presenting two quantitative outcomes. The columns represent the segmentation results of the model.

Figure 6 presents a quantitative display of the segmentation results of the comparative models across four different scenes. In the first row, under the wood scene, the model effectively segments the liquid and combined areas, while the K-Net model shows relatively poor segmentation results. In the tile scene (third row), the model struggles with segmenting fine cracks, resulting in discontinuities; in contrast, our PDeT model performs significantly better. In the hazelnut scene (fifth and sixth rows), the results indicate that K-Net’s segmentation is subpar, while other models demonstrate notably superior performance. In the Metal Nut scene, all models are capable of effectively identifying anomalous defects, showcasing their robust segmentation capabilities.

These results underscore the practical applicability of our model, especially in diverse industrial contexts. By incorporating deformable self-attention and semantic aggregation modules, we not only enhance the geometric distinction capabilities but also refine the representation of semantic features. The introduction of these modules facilitates efficient collaboration between the encoder and decoder, ensuring precise feature transmission and gradual reconstruction, thereby allowing the PDeT to exhibit greater adaptability and stability in real-world applications.

## 5. Conclusions

Our research leverages image sensors for defect detection in photovoltaic panels. Compared to traditional electrical sensor-based methods, such as open-circuit voltage and short-circuit current measurements, we employ electroluminescence (EL) imaging to capture high-resolution, two-dimensional signals, significantly enhancing defect detection accuracy. Additionally, the deep learning techniques incorporated in our study not only improve the perception of defects in photovoltaic panels but also pave the way for advancements in intelligent recognition systems. To further advance this field, we have successfully proposed a Progressive Deformable Transformer for photovoltaic panel defect segmentation, which enhances the segmentation of defects in solar panels. By incorporating deformable self-attention and a semantic aggregation module, we not only improved the ability to differentiate geometric shapes but also refined the representation of semantic features. Furthermore, the introduction of these modules facilitates efficient collaboration between the encoder and decoder, ensuring precise feature transfer and progressive reconstruction. Researchers can flexibly adjust the model based on practical application needs to optimize its performance in specific scenarios. This achievement has significant implications not only for photovoltaic defect segmentation but also provides insights and exploration avenues for research in other industrial defect segmentation fields. The comprehensive experimental results indicate that our method achieved an mIoU of 88.41% on the photovoltaic dataset, outperforming existing segmentation algorithms. Additionally, the network demonstrated comprehensive and high-precision recognition capabilities across four scenarios in the MVTec-AD industrial dataset. By assisting companies in implementing rigorous quality inspections, our approach enhances product quality and economic benefits, promoting the development of sustainable energy systems. By leveraging defect information, photovoltaic manufacturers can optimize production processes, reduce resource waste, and transition to more sustainable manufacturing.

## Figures and Tables

**Table 1:** Ablation study on the application of deformable attention at different stages. ✗ and ✓ indicate whether DTB is used in the stage.

Stage #/Deformable Attention				mIoU
**Stage 1**	**Stage 2**	**Stage 3**	**Stage 4**	
✗	✗	✗	✗	85.27
✗	✗	✗	✓	85.05
✗	✗	✓	✓	88.22
✗	✓	✓	✓	86.17
✓	✓	✓	✓	88.41

**Table 2:** The results of the ablation experiment for the SAB module in the encoding structure. The check mark (✓) signifies the utilization of the SAB, whereas the cross (✗) indicates its exclusion.

Stage #/SAB			mIoU
**Stage 1–Stage 2**	**Stage 2–Stage 3**	**Stage 3–Stage 4**	
✗	✗	✗	87.25
✓	✗	✗	87.87
✓	✓	✗	88.37
✓	✓	✓	88.41

**Table 3:** A comparison of the experimental results with other Decoder heads.

Decoder Head	IoU		mIoU
	**Background**	**Defect**	
FPN head [27]	99.98	74.52	87.25
UPer head [28]	99.98	75.36	87.67
PDeT head (ours)	99.98	76.85	88.41

**Table 4:** Comparison of experimental results of various indicators with other segmentation networks.

Method	Params	FLOPs	FPS	Precision	Recall	F1	mIoU
EMANet [29]	42.08M	263.17G	15.37	34.03	40.53	37.00	61.78
STDC [30]	8.57M	13.24G	78.05	64.88	51.99	57.72	70.02
DDRNet-S [31]	7.72M	9.267G	143.24	68.20	58.62	63.50	73.00
K-Net [32]	62.29M	285.92G	5.99	33.69	82.87	47.91	74.65
DNLNet [33]	50.1M	312.25G	5.75	58.91	79.89	67.82	75.82
CCNet [34]	49.81M	313.09G	12.97	65.29	79.81	71.83	78.15
ANN [35]	46.22M	289.28G	14.00	72.78	78.24	75.41	80.20
DMNet [36]	53.16M	305.64G	13.15	73.95	78.65	76.23	80.61
PIDNet-L [37]	36.93M	53.83G	26.23	71.70	81.40	76.25	80.79
ISANet [38]	37.69M	233.42G	15.37	81.46	75.67	78.46	81.84
GCNet [39]	49.62M	308.89G	13.85	76.93	80.35	78.60	82.51
SIIF [3]	58.72M	75.74G	25.09	79.80	83.03	81.38	84.30
PDeT (our)	104.15M	200.22G	10.49	90.12	89.37	89.66	88.41

**Table 5:** Comparative experimental results of the model across the four scenes: Hazelnut, Metal Nut, Tile, and Wood. All values shown in the table represent the mIoU for assessing the model’s recognition performance in each scene.

Method	Hazelnut	Metal Nut	Tile	Wood
K-Net [32]	76.06	95.29	92.81	74.17
UPerNet [28]	93.57	96.6	95.44	88.35
GCNet [39]	93.27	95.32	95.07	87.93
SIIF [3]	94.13	95.22	92.95	87.86
PDeT (our)	94.34	95.32	92.46	88.54

## Data Availability

The original contributions presented in this study are included in the article; further inquiries can be directed to the corresponding author.

## Abbreviations

The following abbreviations are used in this manuscript:

mIoU	Mean Intersection over Union
IoU	Intersection over Union
mAcc	Mean Accuracy
